# Co-morbid diabetes mellitus in cancer patients undergoing treatment: a case series and perspective

**DOI:** 10.3389/fonc.2025.1615491

**Published:** 2025-07-15

**Authors:** Aisiri H. U. V., Maanas R. Rao, Vinod K. Ramani, Radheshyam Naik

**Affiliations:** ^1^ Department of Biotechnology, Rashtreeya Vidyalaya (RV) College of Engineering, Bangalore, India; ^2^ Preventive Oncology, Sammprada Cancer Hospital, Bangalore, India; ^3^ Medical Oncology, Sammprada Cancer Hospital, Bangalore, India

**Keywords:** chemotherapy, diabetes mellitus, hyperglycemia, hyperinsulinemia, neoplasm

## Abstract

**Introduction:**

Diabetes mellitus (DM) is frequently diagnosed among cancer patients on treatment, which suggests a concomitant underlying mechanism. DM is found among 8 to 18% of cancer patients. The initiation of cancer treatment is linked with the onset of diabetes. This study aims to assess the variation in blood glucose levels of cancer patients during the course of their treatment.

**Methods:**

A hospital record based cross-sectional study was conducted from November 2024 to January 2025, among 50 cancer patients on treatment at Sammprada cancer hospital, Bangalore, India. From the initial 50 cancer patients, a series of 11 cases with large variation in blood glucose levels were included in the study. These include new onset or old cases of diabetes, with blood glucose levels measured across 6 timeframes during the course of cancer treatment.

**Results:**

For the initial 50 patients, the measure of central tendency seemed regressed to the mean. Hence, the design of study was changed from cross-sectional to a case series. For the series of 11 patients all of whom had advanced stage cancer, 5 patients (2 males, 3 females) had history of diabetes and 2 female patients were incident cases of diabetes (1 each in endocrine and cervical cancer). Also, the treatment for diabetes was revised or newly initiated for a total of 5 patients (4 females, 1 male).

**Conclusion:**

Among the 11 cancer patients, 5 had history of DM and 2 were incident cases of DM. Healthcare providers should regularly screen the levels of blood glucose during the continuum of cancer care and monitor the change in trend.

## Introduction

Diabetes mellitus (DM) is frequently diagnosed among cancer patients on treatment, which suggests a concomitant underlying mechanism. Evidence shows that hyperinsulinemia is associated with pathological conditions such as insulin resistance, obesity, inflammation and cancer (1). Globally, ~26.9% of people aged >65 years have diabetes, and 60% of this age-group tend to have cancer (2). DM is found among 8 to 18% of cancer patients (2). The incidence of diabetes and pre-diabetes is significantly higher among cancer patients when compared with normal individuals (2). The initiation of cancer treatment is linked with the onset of diabetes (2). Zhang A.M.Y et al (1) report the association between hyperinsulinemia and increased risk of cancer incidence and mortality. However, the ORIGIN trial did not report an increased risk of cancer among diabetic patients using exogenous basal insulin ‘glargine’ (hazard ratio: 1.00) (4).

Evidence suggests the bi-directional relationship between cancer and DM. The risk of developing cancer is high among patients with DM and hypertension (HTN) when compared with the general population (3). The increased risk of cancer among diabetes patients is due to the underlying hyperinsulinemia, which increases the levels of free and bioactive insulin-like growth factor (IGF-1). Insulin itself belongs to a family of growth factors, whose other members include IGF-1 and IGF-II (4). Apart from its metabolic effects, insulin has important mitogenic effects (4). There is a dearth of randomized trials for demonstrating improved cancer-related outcomes among patients treated for hyperglycemia or for deriving evidence on optimal glucose targets during cancer therapy. Hence, this study is conducted with an aim to assess the variation in blood glucose levels of cancer patients during the course of their treatment.

The increased consumption of glucose by cancer cells is explained by the Warburg effect, which is the principle used in PET scans (5). Warburg initially described the elevated lactate within cancer cells, which indicates a switch in glucose metabolism from aerobic to anaerobic utilization (6). The likely variables which contribute to the elevation in levels of blood glucose include host, biological and treatment characteristics (7). Zhang A.M.Y et al (1) report the strong link between breast cancer and either obesity or diabetes among postmenopausal women. However, during pre-menopause each of obesity and diabetes are associated with a decreased risk for breast cancer (1). Type 2 DM is implicated in the risk or mortality with breast cancer unlike type 1 DM. This implies the causal role played by hyperinsulenima or hyperlipidemia but not hyperglycemia, which is the defining pathophysiological difference between type 2 and 1 DM in their association with breast cancer (1). But, Rabia K.S et al (4) report that the association between cancer and diabetes does not stratify DM distinctly as types 1 and 2. Zhang A.M.Y et al (1) also report the evidence from experimental mouse models on the contribution of hyperinsulinemia to carcinogenesis. The raised level of endogenous insulin promotes the development of esophageal and breast cancers as well as an increase in lung metastases (1).

Cancer patients treated with ICI therapy may trigger autoimmune diabetes across 1 to 2% of cases on such regimen (5). The pathology includes permanent insulin dependent DM, and can be triggered even beyond 6 months of treatment completion (5). Also, immune checkpoint inhibitor (ICI) treatment might worsen the glucose levels among known diabetics and thus warrants continuous monitoring. The anti-tumor effect of ICI is elicited by enhancing the host immune system which could eliminate various types of cancer cells. However, excessive activation of the immune system could cause adverse events which are either concurrent with treatment or late in onset. Corticosteroids as a component of cancer treatment regimen are likely to reduce the insulin sensitivity and subsequent onset of hyperglycemia and diabetes, as well as influence the lipid metabolism (2). Evidence shows that TKIs and mTOR inhibitors interfere with glucose metabolism (5). This risk also exists for other forms of cancer treatment such as L-asparaginase (chemotherapy), calcineurin inhibitors (immunosuppressive agents) and total body irradiation therapy (2).

Evidence indicates that hyperglycemia contributes to a malignant phenotype of cancer, which includes proliferation, inhibition of apoptosis, metastasis, perineural invasion and resistance to chemotherapry (5). The complications of hyperglycemia are due to the increased production of reactive oxygen species (ROS) from mitochondria. Such elevated levels of ROS leads to cellular DNA mutations, which is pathognomonic for multistage carcinogenesis (5). DM also affects the physical health and quality of life of cancer patients (8). It can enable an aggressive clinical course of cancer by impairing the immune function of the patient, as well as causing physiological distress and decreasing the quality of life (2). DM can significantly increase the mortality among cancer patients (2). The rationale for this study stems from the need to recognize the risk of elevated levels of blood glucose among cancer patients, and design appropriate strategies for integrating diabetes and cancer care (2).

## Methods

### Study design and setting

A hospital record based cross-sectional study was conducted from November 2024 to January 2025, among 50 cancer patients on treatment at Sammprada cancer hospital, Bangalore, India. Among them, a series of 11 cases with large variation in blood glucose levels were included in the study. These include new onset or old cases of diabetes.

### Variables

The diagnosis of diabetes was done as per the American Diabetes Association (ADA) guidelines, which mentions a cut-off venous random blood glucose (RBS) value >200 mg/dl and presence of symptoms of diabetes. Patients with previous diagnosis of diabetes or current use of anti-diabetes medication were also included.

### Data collection procedure

Secondary data was collected by trained Interns from the Oncology department under the supervision of Principal Investigator and Medical oncologist.

## Results

**Figure 1:**
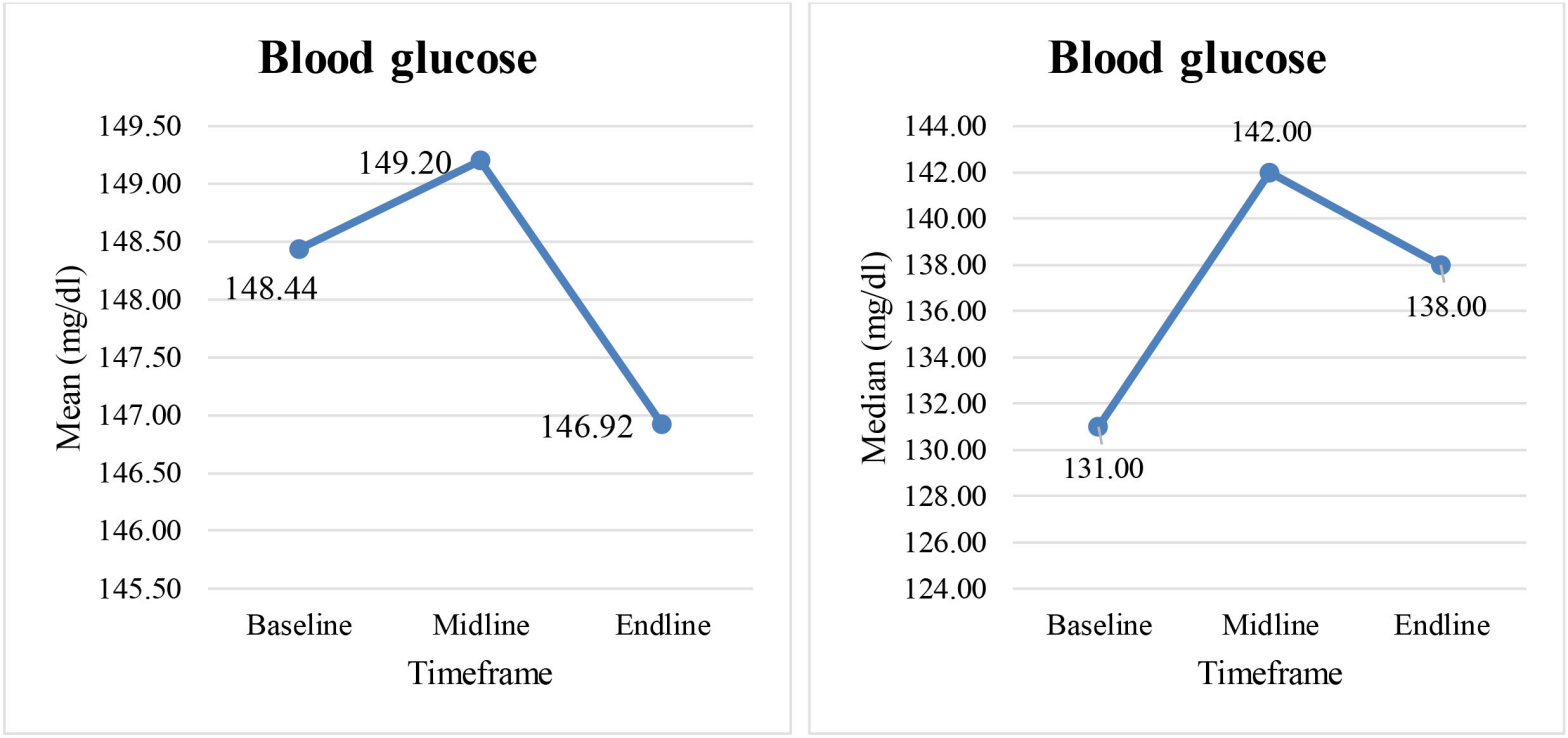
Plot of mean and median RBS values across 3 timeframes for 50 cancer patients.

For the initial group of 50 cancer patients, random blood glucose values were analyzed across 3 timeframes (baseline, midline, endline) of cancer treatment. **Figure 1** depicts the measure of central tendency, where-in mean glucose values are more than the median values thus inferring right skewing of data for all the 3 timeframes. The normal values of RBS tend to pull the abnormal values closer to the center of data distribution. Thus mean or median are not ideal statistics, as they currently appear less extreme than when the abnormal values were not regressed. Hence, the study design was changed from cross-sectional to a case series. The study subjects thus include 11 patients with abnormal blood glucose values, teased from the initial group of 50 cancer patients.

**Table 1** shows the clinicosocial profile of 11 cancer patients with abnormal blood glucose values, measured during the six timeframes of cancer treatment. Across the timeframe, the values were distributed around the cut-off of ≤200 mg/dl (as per ADA criteria) (9) with a few outliers. Among the 11 patients who also presented with advanced stage cancers, 5 patients (2 males, 3 females) had history of DM and 2 female patients (1 each in endocrine and cervical cancer) were incident cases of DM. Also, the DM treatment was revised or newly initiated for a total of 5 patients (4 females, 1 male).

**Table 1:** Clinicosocial profile of cancer patients with abnormal blood glucose values.

Sl. No.	Patient	Sex	Age (yrs)	Type of cancer	Anti-cancer treatment	h/o DM	Current DM medication	New drug for DM*
1	A	F	79	Cholangio-carcinoma	Cisplatin, Gemcitabine, Durvalumab,	No	–	–
2	B	F	76	B cell lymphoma	Rituximab, Cyclophosphamide, Doxorubicin, Vincristine, and Prednisone	Yes	T.Intaglip 50 mg, T.Glime 2 mg	–
3	C	F	60	Breast	Nab-paclitaxel, Gemcitabine	No	–	–
4	D	F	63	Endocrine	Sandostatin LAR, Bevacizumab	No	–	T.Glycomet 500 mg
5	E	F	64	Breast	Eribulin mesylate, Capecitabine	Yes	T.Ubermet 500 mg	T.Sitagliptin 50 mg, T.Diapride M 1 mg
6	F	F	58	Breast	Carboplatin, Nab-paclitaxel	No	–	–
7	G	M	80	Pancreas	Gemcitabine, Nab-paclitaxel	No	–	–
8	H	M	80	Lung	Carboplatin, Pemetrexate, Bevacizumab,	Yes	T.Dynaglipt M 20/500 mg Inj.Basalog insulin 8 units	Continue previous medication
9	I	F	38	Ovary	Carboplatin, Nab-paclitaxel	Yes	T.Metformin 250 mg	T.Glycomet 250 mg
10	J	F	53	Cervix	Carboplatin, Nab-paclitaxel	No	–	T.Glycomet trio 2 mg, Inj.Lantus insulin, T.Glyciphage PG2,
11	K	M	63	Synovial cell	Ifosfamide, Adriamycin	Yes	T.Glyciphage PG, Inj.Lantus insulin	T.Glycomet trio, Inj.Lantus insulin

*introduced for the management of DM.

**Figure 2:**
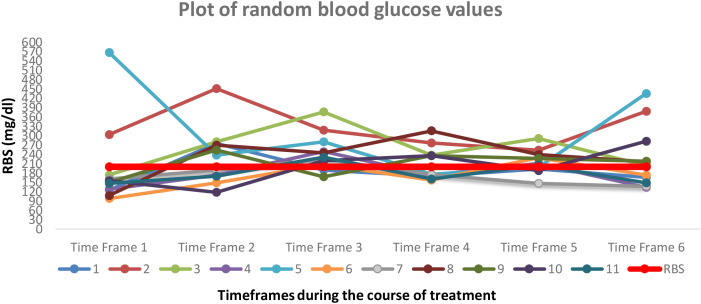
Plot of RBS for 11 patients at 6 timeframes during the course of cancer treatment.

**Figure 2** depicts the variation in random blood glucose values for 11 patients during the course of cancer treatment. As per the ADA criteria, RBS cut-off value of 200 mg/dl is depicted by the red line in the center of the plot. The graph shows the trend of blood glucose values and depicts a profound variability across the 6 timeframes.

## Discussion

The 11 study subjects in the case series include cancer patients with large variations in blood glucose levels, measured across 6 timeframes during the course of cancer treatment. Among them, 5 patients had history of DM and 2 patients were incident cases of DM. The presence of co-morbid DM among cancer patients could be stratified as patients with history of DM, previously unknown DM and cancer treatment induced DM (5). Pliszka M et al (10) suggests the increased risk of DM among patients with liver (SRR: 2.01, 95% CI: 1.61-2.51), pancreatic (RR: 1.94, 95% CI: 1.66-2.27), colorectal (RR: 1.26, 95% CI: 1.2-2.31), esophagus (SRR: 1.3, 95% CI: 1.12-1.5), kidney (RR: 1.42, 95% CI: 1.06-1.91), bladder (RR: 1.24, 95% CI: 1.08-1.42), breast (RR: 1.2, 95% CI: 1.12-1.28) and endometrial (RR: 1.65, 95% CI: 1.5-1.81) cancers. The burden of elevated blood glucose among cancer patients could also be due to the shared risk factors such as increasing age, obesity, physical inactivity, poor diet, alcohol consumption and smoking use (2). Treatment with certain chemotherapies, biological agents and glucocorticoids are likely to precipitate hyperglycemia among cancer patients (7).

Hyperglycemia in cancer patients is associated with a severe course of cancer, along with adverse events such as neutropenia, infections and increased mortality (6). The mechanisms of hyperglycemia include an increase in insulin resistance and a reduction in β cell function. Another method comprises the inhibition of glycogen synthesis, promotion of glycogenolysis along with impeding the peripheral uptake of glucose (6). Zhang A.M.Y et al (1) report the strong association between hyperinsulinemia and chronic low grade inflammation. Insulin and the downstream (AKT/PI3K) signaling have important effects on both the cytotoxic and regulatory T cells, including innate immune cells (macrophages). In addition, cancer cachexia can cause impaired glucose tolerance through various mechanisms (2).

Wolde H.F et al (2) report the proportion of elevated blood glucose among cancer patients as 73.4% (95% CI 68.8, 77.6), with prediabetes constituting 56.8% (95% CI 51.7, 61.7) and diabetes comprising 16.7% (95% CI 13.3, 20.8). Among the identified DM cases, 40.6% were newly diagnosed and the remaining were known DM on medication. Stage IV cancer was associated with a higher proportion (75.5%) of elevated blood glucose when compared with Stage 1 (71.7&). Alcohol consumption was associated with increased odds of elevated blood glucose levels in cancer patients (AOR: 1.96; 95% CI: 1.11, 3.46). Evidence also supports the association between dysbiosis (disruption of gut microbial diversity and function) and an increase in the risk of insulin resistance, diabetes and several types of cancers (4). The mechanisms hypothesized include chronic inflammation, impaired immune function of the host, production of carcinogenic byproducts and disruption of programmed cell death (4).

Osei et al’s (8) study reports the prevalence of hypertension, hyperlipidemia and type 2 DM among colon cancer patients as 52.3%, 53.0% and 15.1% respectively. This data was derived from the ‘All of Us’ research database in the United States, which includes data from the electronic health records (EHR). The prevalence of the 3 disorders among other participants was 21.6%, 22% and 7.9% respectively. The calculated odds ratio of the 3 disorders are 4.05 (95% CI: 3.74-4.4), 4.06 (95% CI: 3.74-4.41) and 2.08 (95% CI: 1.86-2.33) respectively. Colon cancer patients with co-morbid DM had a significantly lower mean score of physical health and quality of life when compared with non-diabetic patients. However, these parameters (physical health and quality of life) were similar for the other 2 co-morbid conditions ‘hypertension’ and ‘hyperlipidemia’.

Research studies on healthy individuals report the average levels of insulin measured over a duration of 24 hours as fasting insulin: 60pmol/L and post-meal insuln: 420 pmol/L (1). The proposed cut-off for hyperinsulinemia is fasting insulin >85 pmol/L (12.2 mIU/L), which is adequate to mark the condition of metabolic syndrome. Zhang A.M.Y et al (1) report the average level of fasting insulin as 140 pmol/L and post-meal insulin as 840 pmol/L for individuals with obesity. The biochemical reasoning behind insulin driven obesity includes the profuse signaling in adipocytes which leads to excess fat accumulation (1). Given the catabolic nature of cancer, caution is advised for using anti-diabetic medication such as metformin, SGLT2 inhibitors and GLP-1 receptor agonists which have known effects of weight loss. Insulin could be the alternative choice given its anabolic effect (6).

Chemotherapy drugs likely to cause hyperglycemia among patients without DM include cisplatin, 5-fluorouracil, anthracyclines, as well as chemoradiation treatment (6). The proposed mechanisms include an induction of an inflammatory state or the direct metabolic effects on tissues such as skeletal muscles which are vital for glucose homeostasis (11). Other mechanisms include damage to pancreatic β cells, hormonal deficiencies, reduced physical activity, weight gain, insulin resistance, changes in lipid metabolism and inflammatory mediators (12). The chemotherapy drug L-asparaginase is likely to cause impaired pancreatic β cell function and pancreatitis, thus inducing hyperglycemia. Novel targeted agents are likely to block the IGF-1 receptor thus enabling the release of growth hormone. Other agents which inactivate cell proliferation pathways such as Ras/MAPK/extracellular regulated kinase (ERK) and PI3K/AKT/mTOR, tend to interrupt the intracellular response to insulin (13). Corticosteroids predominantly affect the postprandial levels of glucose, and levels of fasting plasma glucose may be normal in these patients (6). The reasons implicated include an increase in insulin resistance, reduction of insulin secretion and an escalation in the hepatic glucose output.

Duan W et al (6) report the role of hyperglycemia on cancer proliferation, where-in proliferation assays reveal that high levels of glucose (11 Mmol/L) and insulin (100 ng/mL) have promoted the growth of following tumor cell lines: HT29 (human colon carcinoma), SW480 (human colorectal carcinoma), MCF-7 (human breast adenocarcinoma), MDA MB468 (human breast adenocarcinoma), PC3 (human prostate cancer), and T24 (human bladder carcinoma). The authors report an increase in the expression of collagen receptors under hyperglycemic conditions, as well as the integrin-linked kinase regulating cellular processes such as growth and proliferation. Also, hyperglycemia was reported to increase the proliferation of breast cancer cells by upregulating cdk2 (cyclin dependent kinase 2) and cyclin D1, which in-turn accelerate the cell cycle progression. In pancreatic cancer cell lines, glucose concentration tends to alter the cell proliferation in a concentration dependent manner through the expression of GDNF (glial cell line derived neurotrophic factor) and RET (tyrosine kinase receptor). Glucose metabolism in cancer cells tends to protect it from cytochrome C-mediated apoptosis.

The existing metabolic compensation in chronic diabetics may be further worsened by anti-cancer therapy, which exacerbates the subsisting organ damage (6). In cancer patients with DM, the cardiovascular risk and complications should be factored for determining the treatment regimen. The adverse effects of chemotherapy also include worsening of renal function and neuropathic complications. Given the possibility of renal complications, preventing dehydration should be prioritized for avoiding acute kidney injury. Chemotherapy drugs such as platinum derivatives and taxanes are likely to cause peripheral neuropathy which may persist for up to 2 years after treatment (6). Evidence shows that targeted therapies (kinase inhibitors), monoclonal antibodies, poly (ADP-ribose) polymerase, phosphoinositide 3-kinase (PI3-K), and mTOR inhibitors tend to exert detrimental effects on glucose and lipid metabolism, as well as on blood pressure and the cardiovascular system (6).

Rabia K.S et al (4) inform that for cancer patients with co-morbid diabetic nephropathy, dose adjustments are necessary for a few anti-cancer drugs This includes ‘cisplatin’ used for the treatment of germ cell tumors and ‘capecitabine’ used for gastrointestinal cancers. The dose adjustments are mandatory among diabetic patients with pre-existing heart disease as seen with adjuvant trastuzumab for HER2-positive breast cancer. This drug is contraindicated among patients with left ventricular dysfunction. Chemotherapy induced cardiotoxicity manifests as arrhythmias, cardiomyopathy and vascular thrombosis, further leading to myocardial infarction. Such complications are further hastened with other forms of treatment such as targeted, endocrine and radiation therapy. Cancer patients with complications of diabetic neuropathy mandate the restricted usage of drugs such as platinum compounds (cisplatin, oxaliplatin), vinca alkaloids (vincristine), taxanes (paclitaxel), thalidomide and bortezonib. However, such dose adjustments carry the risk of tumor recurrence.

Limited glycemic control in cancer patients leads to increased pain, asthenia and a higher incidence of treatment related toxicities such as nausea, vomiting, loss of appetite, diarrhea and weight loss. The resultant malnutrition and loss of skeletal muscle mass causes a decline in functional status, which warrants appropriate management (6). The glycemic targets should be individualized, which prevents the possibility of hyper- or hypoglycemia. In this regard, factors which need to be considered include age, performance status, life expectancy, disease stage, co-morbidities and risk of hypoglycemia. A balanced diet will facilitate glycemic control and an improvement in the patient’s energy levels. Regular exercise benefits both DM and cancer, through maintaining the muscle mass and delaying the risk of cancer associated cachexia.

Patients on anti-cancer treatment who develop high blood glucose levels should be regularly monitored for levels of insulin and C-peptide, pancreatic amylase/lipase, anti-islet cell autoantibodies, anti-glutamic acid decarboxylase autoantibodies, anti-insulin antibodies and urine/capillary ketones (6). Patients who are already diabetic should be regularly monitored for levels of fasting/post-prandial plasma glucose, glycated hemoglobin (HbA1c), low density cholesterol (LDL-C), triglycerides, and values of blood pressure. When using HbA1c for evaluating the glycemic status of anemic patients and those with hematological malignancies, there exists a risk of inaccurate results given the need of blood transfusion.

Given the underlying mechanisms for the development of hyperglycemia, it is vital to choose the appropriate anti-diabetic medication. Insulin resistance due to kinase/mTOR inhibitors or corticosteroids needs to be treated with insulin sensitizers, and insulin deficiency due to immunotherapy or post-pancreatitis/pancreatic cancer needs mandatory supplementation of insulin (6). The article builds the perspective for an imperative screening of DM in cancer patients before initiating anti-cancer treatment. This includes assessment of diabetic complications, nutritional status and metabolic homeostasis.

## Limitations

- The study design is record based cross-sectional in nature, which precludes any inference on causation. The sample size of the study is ‘50’, however a prospective cohort study with a larger sample size will enable the effective study of outcomes. Since HbA1c was not used as a diagnostic criteria for pre-diabetes or diabetes, the morbidity could be overestimated,
- The study did not assess the association of socio-demographic characteristics of patients such as age, sex, geography of residence, religion, occupation and income. Also, a structured questionnaire was not used to assess the behavioral characteristics such as cigarette smoking, alcohol consumption, chewing smokeless forms of tobacco, type of diet and physical activity. Ideally, the cancer related characteristics such as type and stage, duration of disease and treatment, type of treatment delivered and presence of metastasis need to be factored,
- The study did not correlate other physical measurements such as weight, height, waist circumference (WC) and blood pressure (BP).

## Conclusion

This study shows a large variation in blood glucose levels among the series of 11 patients during the course of their cancer treatment. The morbidity of DM among cancer patients needs to be further probed. Healthcare providers should regularly screen for the levels of blood glucose during the continuum of cancer care, and monitor the change in trend. Poorly controlled DM and/or hypertension can significantly influence the management of cancer (14). It is important to demonstrate the implications of hyperglycemia on the response to cancer treatment and subsequent health outcomes. In the future, prospective cohort studies should be designed for assessing the temporal relationship between cancer and DM. The treatment plan for patients on anti-cancer therapy should include algorithms for periodically monitoring their vital signs, laboratory values and electrocardiogram (ECG). The study highlights the need for regular screening of blood glucose and blood pressure among cancer patients, and their optimal management for improving the patient outcomes.

## Data Availability

The data analyzed in this study is subject to the following licenses/restrictions: The dataset can be accessed by writing to the corresponding author. Requests to access these datasets should be directed to Vinod K Ramani, vinodramani77@gmail.com.
